# Long-Time Exposure to Violent Video Games Does Not Show Desensitization on Empathy for Pain: An fMRI Study

**DOI:** 10.3389/fpsyg.2017.00650

**Published:** 2017-05-02

**Authors:** Xuemei Gao, Wei Pan, Chao Li, Lei Weng, Mengyun Yao, Antao Chen

**Affiliations:** ^1^Faculty of Psychology, Southwest UniversityChongqing, China; ^2^Key Laboratory of Cognition and Personality, Ministry of Education, Southwest UniversityChongqing, China

**Keywords:** violent video games, violence, empathy, empathy for pain, fMRI

## Abstract

As a typical form of empathy, empathy for pain refers to the perception and appraisal of others’ pain, as well as the corresponding affective responses. Numerous studies investigated the factors affecting the empathy for pain, in which the exposure to violent video games (VVGs) could change players’ empathic responses to painful situations. However, it remains unclear whether exposure to VVG influences the empathy for pain. In the present study, in terms of the exposure experience to VVG, two groups of participants (18 in VVG group, VG; 17 in non-VVG group, NG) were screened from nearly 200 video game experience questionnaires. And then, the functional magnetic resonance imaging data were recorded when they were viewing painful and non-painful stimuli. The results showed that the perception of others’ pain were not significantly different in brain regions between groups, from which we could infer that the desensitization effect of VVGs was overrated.

## Introduction

Recently, the society has witnessed the rapidly development in video game industry. A non-negligible issue is that most of the video games contain violent content (Yoon and Somers, 2003), which could be harmful to the players, even jeopardize the public safety. The relationship between exposure to media violence and its potential negative effects has been the subject of social, political, and scientific attention for decades. Playing violent games may heighten aggressive behavior, cognition, and affection; increase physiological arousal and hostility; and decrease the probability of helping others (e.g., Anderson and Bushman, 2001, 2002; Bushman and Anderson, 2001, 2009; Anderson et al., 2004, 2008; Gentile et al., 2004; Bartholow et al., 2005; Bushman and Huesmann, 2006). Playing violent video games (VVGs) also has a desensitizing physiological effect (Carnagey et al., 2007), and may also be associated with non-violent delinquent behaviors (Anderson and Dill, 2000; Ferguson and Kilburn, 2009; Desai et al., 2010; Gunter and Daly, 2012), such as cheating, skipping school, stealing, and substance abuse. Previous researches have investigated the relationship between empathy and exposure to video game violence. It has been suggested that exposure to VVG was associated with lower empathy (Funk et al., 2004; Anderson et al., 2010). However, the existing researches concerning desensitization effects of VVG are not inconsistent. For example, Ferguson and Kilburn (2010) conducted a meta-analysis and the results suggested that VVGs were not significantly associated with aggression, neither with prosocial behavior (Jerabeck and Ferguson, 2013; Elson and Ferguson, 2014; Tear and Nielsen, 2014). Instead, some of the VVGs could even increase the cognition abilities such as object tracing, spatial discrimination, and central attention (Green and Bavelier, 2006, 2007). Even the inconsistent researches are not quite much, given the publication bias and the validity of behavioral investigations, the null results of previous researches should also pay attention to. These indicated that the long-time effect of VVGs should be carefully examined further.

Empathy is a crucial component of human emotional experience and social interaction (Bernhardt and Singer, 2012), which is vital to our everyday communication and survival in a social environment (Fan et al., 2011). Usually, empathy refers to the capacity to understand and share the emotional and affective states of another person in relation to oneself (Decety and Jackson, 2004; Singer et al., 2006; Hein and Singer, 2008; Guo et al., 2012, 2013). The capacity for empathy allows us to understand others’ emotions, motivations, and behaviors, which help us to decide what we can do. Empathy for pain is a typical form of empathy. When witnessing other people suffering in pain, the observers often show compassion, sympathy and care-giving to them (Goubert et al., 2005). The empathy for pain is attracting increasing attention because of its survival value embodied in the capacity that positively correlates to prosocial behavior and behaviors conforming to our social norms (Hoffman, 2008).

There are a growing number of functional magnetic resonance imaging (fMRI) studies that focus on empathy for pain. Research demonstrates that the first-hand experience of pain and the observation of others in pain activate similar neural circuits. These neural circuits consist of areas encoding different dimensions of pain perception. The primary and secondary somatosensory cortex mainly subserve the sensory-discriminative dimension of pain (e.g., Bushnell et al., 1999; Avenanti et al., 2005; Valeriani et al., 2008; Akitsuki and Decety, 2009), whereas the supplementary motor area (SMA), cerebellum, insula, anterior cingulate cortex (ACC), and the anterior mid-cingulate cortex (aMCC) mainly subserve the affective-motivational dimension of pain (e.g., Singer et al., 2004; Danziger et al., 2006; Gu and Han, 2007; Lamm et al., 2007; Akitsuki and Decety, 2009). These two dimensions are highly correlated (Decety, 2011). There are also brain regions encoding the cognitive-evaluative dimension of pain, such as the temporoparietal junction (TPJ) and the orbitofrontal cortex (OFC), which are involved in social interaction, intention, and belief (e.g., Walter et al., 2004; Amodio and Frith, 2006; Moll and de Oliveira-Souza, 2007). Other regions, like the amygdala, thalamus, and periaqueductal gray (PAG) may also be activated when watching others in pain (e.g., Phelps et al., 2001; Adolphs, 2002; Winston et al., 2003). Furthermore, empathy or empathy-related neural networks may interact with (and be modulated by) the activity of other neural networks relevant for social cognition, such as mentalizing, cognitive control, and emotion regulation (Bufalari and Ionta, 2013). Based on these findings, it is apparent that pain empathy is associated with cognitive and emotional regions such as TPJ, OFC, and ACC, which were vital in cognitive control and moral judgment (Molenberghs et al., 2015). We could speculate that lack of empathy for pain in others may lead to terrible consequences, not only to the individuals themselves, but also the whole society.

Long-time exposure to VVGs may blunt this capacity and result in undesirable consequences. In this research, we mainly focused on the relationship between long-term exposure to video game violence and empathy to investigate the negative effects of media violence on empathy, especially empathy for pain for others. According to former researches, desensitization to video game violence may be a core factor to low capacity of empathy for pain in others. It has been repeatedly proved that longtime exposure to video game violence leads to desensitization, which refers to impaired emotional response to negative feelings coupling with aggressive consequences. This may cause numb senses of knowing the pain and suffering of others which may result in low empathy for others’ pain. A negative correlation was confirmed between long-term video game violence exposure and empathy (Funk et al., 2004; Bartholow et al., 2005; Krahé and Möller, 2010). There are also neuropsychological evidences to support this argument. Playing VVGs can affect some regions or neural circuits of the frontal lobe (e.g., Davidson et al., 2000; Mathiak and Weber, 2006; Wang et al., 2009), and this may affect the response of gamers to emotional stimuli (Kühn et al., 2011). Similar results were found by Gentile et al. (2016) that long time exposure to VVGs may lead to suppression in regions relating to emotional response regions and cognitive regions and abnormal activities in cognitive control regions (Gentile et al., 2016). Moreover, Montag et al. (2012) assumed that due to a frequent confrontation with violent scenes, the first-person-shooter-video-gamers might have habituated to the effects of unpleasant stimuli, resulting in lower brain activation. Coincidentally, Guo et al. (2013) explored participants’ empathic responses after short-term exposure to violent videos, using fMRI. They found that short-term exposure to media violence reduced the activation of the aMCC and insula, and proposed that exposure to media violence had a desensitizing effect. These indicated that longtime exposure to media violence is associated with empathy for pain in others. In addition, it also should be noted that extant literature still exist some inconsistency between VVGs and desensitization effect, which needs further exploration.

What’s more, there currently is few fMRI research exploring the relationship between exposure to video game violence and pain empathy. This kind of research can help us to understand the neural mechanism of empathy and to identify the influence of violent games on the brain. Based on past research on empathy among healthy participants (e.g., Akitsuki and Decety, 2009; Fan et al., 2011, 2014; Lamm et al., 2011) and certain types of groups (e.g., Mathews and MacLeod, 2005; Decety et al., 2009), as well as research on the affective processing and empathy among violent video gamers (e.g., Anderson et al., 2010; Barlett and Anderson, 2011; Zhen et al., 2011; Guo et al., 2013), the present study aimed to identify whether video game violence can affect the capacity of empathy for pain, if so, how video game violence can affect the capacity of empathy for pain.

## Materials and Methods

### Participants

All the participants were recruited from the community of Southwest University (Chongqing, China). Participants were selected from approximately 200 undergraduates who completed a video game questionnaire (Anderson and Dill, 2000) that examined their previous game experience. We calculated a score representing their previous game experience and then randomly selected 20 individuals scoring above the 75th percentile and 20 individuals scoring below the 25th percentile, to comprise high and low previous-exposure groups (VG and NGs), respectively. All the participants were males aged 18 to 27 (*M* = 21.17, *SD* = 2.065). All the participants were right-handed and had normal or corrected-to-normal vision. None of them had a history of neurological or psychiatric disorders. All participants gave informed consent before scanning. After the experiment, they were paid for their participation.

### Materials

#### Video Game Questionnaire

The video game questionnaire (Anderson and Dill, 2000) was used to select participants. Participants were asked to list three their favorite video games, indicate the number of hours they played each game in a week, and then rate the violence of their content and graphics (from 1 = not at all to 7 = extremely). Previous game experience was measured by summing the content and graphics ratings for each game, multiplying the sum of the number of hours the game was played each week, and then averaging across the three games. The questionnaire showed good reliability and validity. The internal consistency coefficient was 0.89–0.91, and the Q factor of each factor reached 0.7 (attractive factor: 0.77; violence factor: 0.90; time factor: 0.73).

#### Interpersonal Reactivity Index-China (IRI-C)

Interpersonal Reactivity Index-China (IRI-C) is a 22-item questionnaire for measuring trait empathy. The IRI-C is the Chinese version of IRI (Zhang et al., 2010), and four dimensions were included in this questionnaire: perspective taking, fantasy, empathic concern, and personal distress. IRI-C has been demonstrated to have satisfactory reliability and validity (Zhang et al., 2010; Jiang et al., 2014).

#### Stimuli in the Experiment

**FIGURE 1:**
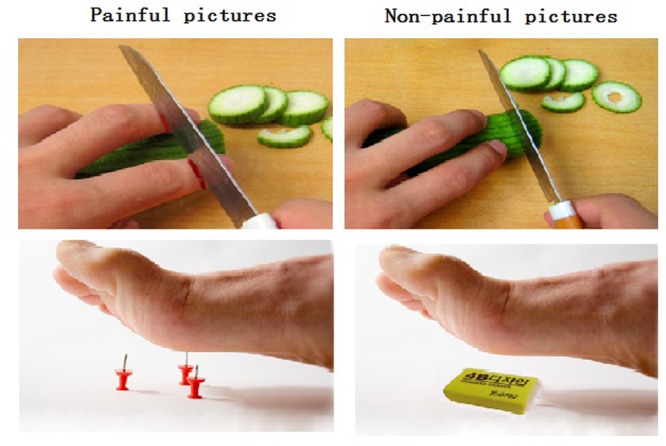
**Illustration of the painful and non-painful pictures. (Left)** The left panel shows examples of non-painful pictures. **(Right)** The right panel shows examples of painful pictures.

Eighty digital color pictures showing people’s hands, forearms, or feet in painful or non-painful situations (40 pictures each) were used as stimuli. All situations depicted familiar events that occasionally happen in our everyday life; the stimuli were similar to those used by Meng et al. (2012). Examples of painful situations depicted a hand cut by a knife and a foot stabbed by pins (**Figure 1**). Non-painful situations were paired with painful situations, without any nociceptive components, such as using a knife to cut cucumbers and a foot touched by an eraser. All pictures were shot from first-person perspectives and edited to the same size. Luminance, contrast, and color were matched between painful and non-painful pictures (Meng et al., 2012).

### Procedure

First, participants were scanned to acquire high-resolution structural images. Then functional images were acquired, while the participants viewing the stimuli displayed on a gray background. The E-prime software (Psychology Software Tools, Inc., Pittsburgh, PA, USA) and a back-projection system were used for presenting the stimuli. All the pictures were randomly presented on the screen and the procedure in each run was exactly the same. Participants were asked to watch each picture carefully and to try to experience the feelings of the persons whose body parts were shown in the pictures. The oddball paradigm was used to ensure that participants viewed the pictures carefully and did not close their eyes. This entailed two kinds of trials: stimulus-only trials and stimulus–response trials.

**FIGURE 2:**
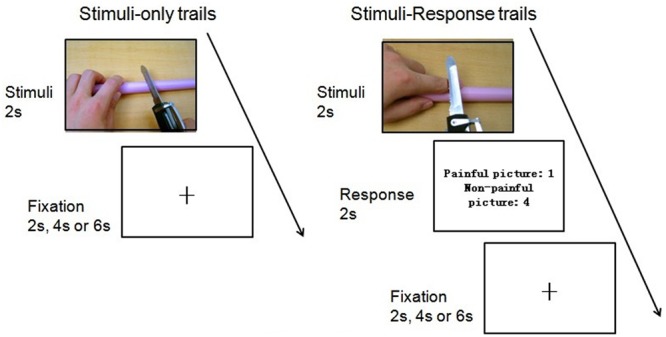
**Flow paradigm of the experiment**.

In stimulus-only trials, each picture was presented for 2,000 ms with jittered inter-stimulus intervals (ISI, lasted for 2,000, 4,000, or 6,000ms), during which a black fixation point was presented against the gray background. Participants were instructed to view the picture carefully and just wait for the next trial. In stimulus–response trials, each picture was presented for 2,000 ms, followed by a response screen showing the following message: “painful picture: 1; non-painful picture: 4.” Participants were instructed to press “1” if they thought the picture was painful, and to press “4” if they thought the picture was non-painful. This screen remained for 2,000 ms. Jittered ISI were used as they were in the stimulus-only trials. Stimulus–response trials made up about 20 percent of all trials (16 trials) in the experiment. The two kinds of trials were randomly presented during the experiment (see **Figure 2**).

All the participants responded in all 17 stimulus–response trials. The mean number of correct answers of all participants was 14.64 (*SD* = 2.13). Participants in the two groups did not differ significantly in their mean number of correct answers (*M*_V G_ = 14.57, *SD* = 2.31; *M*_NG_ = 14.71, *SD* = 2.02). Therefore, both groups viewed the presented pictures equally.

### fMRI Image Acquisition and Analysis

Scanning was performed with a whole-body 3T Siemens scanner (Siemens Magnetom Trio Tim, Southwest University, Chongqing, China). Functional images were acquired using an echo-planar imaging (EPI) sequence and the following parameters: slice number = 32, TR = 2000 ms, TE = 30 ms, flip angle = 90°, matrix size = 64 × 64, slice thickness = 3 mm. Images were acquired using an ascending interleaved sequence with no temporal gap between consecutive image acquisitions. There was one run of functional scanning that was approximately 9 min (270 EPI volumes). A high-resolution structural image was acquired using a T1-weighted, multiplanar reconstruction (MPR) sequence and the following parameters: TR = 1900 ms, TE = 2.52 ms, slice thickness = 1 mm, flip angle = 9°, matrix size = 256 × 256, voxel size = 3 mm × 3 mm × 3 mm.

Data preprocessing was carried out with SPM8 (Statistical Parametric Mapping, Wellcome Department of Imaging Neuroscience, London, UK) implemented in MATLAB 7.0 (The Math Works, Inc., Sherborn, MA, USA). The first five volumes were discarded to allow for T1 equilibration effects. Data preprocessing included slice-timing correction, correction for head motion (realigned to the first volume), normalization, and smoothing using a 6-mm full-width half-maximum isotropic Gaussian kernel. It should be noted that head motions in all participants were corrected and met the criteria with head motion < 3 mm. In this case, three participants were deleted from the analysis.

We then analyzed the neural responses to painful and non-painful stimuli in the VG (PVG and NVG) and in the NG (PNG and NNG). Statistical analyses were performed using the general linear model (GLM) implemented in SPM8. GLMs were estimated using a hemodynamic response function and a high pass filter of 128 Hz, as well as correction for autocorrelations. For the analysis, the six movement regressors of each subject were also included in the design matrix as covariates. The simple main effects of each subject for two types of events (P and NP) were computed by applying ‘1 -1’ contrasts. The four first-level individual contrast images (PVG, NVG, PNG, and NNG) were then analyzed at the second group level adopting the methods of independent samples *t*-test.

Brain activation representing the perception of painful stimuli was defined using the (PVG + PNG) – (NVG + NNG) contrast. The full factorial model was established to identify different brain regions between NG and VG [(PNG - NNG) - (PVG - NVG), (PVG - NVG) - (PNG - NNG)].

## Results

Two participants in the NG did not complete our study so their data were deleted. The data of two participants in the NG and one in the VG were deleted either because of excessive head movements. Hence, the data in our final analysis were collected from 35 participants, including 18 participants in the VG and 17 participants in the NG.

### Behavioral Data

An independent-sample *t*-test of VVG exposure in two conditions was conducted and results were found the difference of familiarity with the VG (*M* = 142.39, *SD* = 18.66) and NG (*M* = 25.62, *SD* = 6.44), *t*(33) = 5.78, *p* < 0.05, suggesting significant difference between VG and NG.

No significant difference between VG (*M* = 49.38, *SD* = 8.57), and NG (*M* = 47.52, *SD* = 10.79) in IRI-C total score, *t*(33) = 0.57, *p* > 0.05, either in each dimension [*t*(33) = 0.11, 0.26, 1.09, and 0.68, *p* > 0.05].

No significant difference between VG (*M* = 72.12, *SD* = 17.49), and NG (*M* = 72.66, *SD* = 25.19) in BPAQ total score, *t*(33) = -0.077, *p* > 0.05, either in each dimension [*t*(33) = -0.034, 0.365, -0.065, and -0.397, *p* > 0.05].

### fMRI Data

**FIGURE 3:**
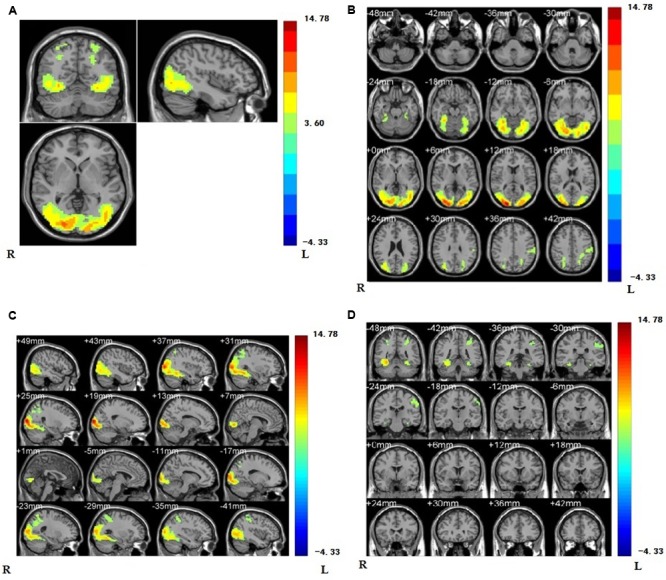
**(A)** Regions showing higher activation are the regions of supramarginal gyrus, lateral middle occipital gyrus, lateral fusiform gyrus, right inferior occipital gyrus, inferior parietal gyrus, middle temporal gyrus, and visual related regions such as V2 compared with non-painful stimuli (*p* < 0.001, Alphasim corrected; *k* > 1361). We have separately MR imaging at the position of oblique-axial **(B)** plane, oblique coronal; **(C)** and sagittalia; **(D)** in regions activated.

As a first step, we contrasted the brain activity of the painful conditions with the non-painful conditions for all the subjects. The results of (PVG + PNG) - (NVG + NNG) showed that when viewing others in pain, regions were activated in the right supramarginal gyrus (rSMG), lateral middle occipital gyrus, lateral fusiform gyrus, right inferior occipital gyrus, inferior parietal gyrus, middle temporal gyrus, and visual related regions such as V2 (see **Figure 3** and **Table 1**).

**Table 1:** Brain regions showing significant activation in lateral middle occipital gyrus, lateral fusiform gyrus, right inferior occipital gyrus, right supramarginal gyrus, inferior parietal gyrus, middle temporal gyrus, and visual related regions such as V2 while viewing painful stimuli compared with non-painful stimuli (*p* < 0.001, Alphasim corrected; *k* > 1361).

Region of activation	Lat.	MNI			*t-*value	*k*
		*x*	*y*	*z*		
Middle occipital gyrus	R	21	–96	12	14.78	483
Middle occipital gyrus	L	–24	–87	12	9.04	683
Fusiform gyrus	R	30	–51	–12	9.90	427
Inferior occipital gyrus	R	33	–75	–9	9.06	216
Fusiform gyrus	L	–30	–63	–12	7.68	357
Supramarginal gyrus	R	33	–54	57	5.18	130
Inferior parietal gyrus	L	–27	–51	48	4.76	187
Secondary visual cortex (V2)	R	36	–87	9	11.75	369

**FIGURE 4:**
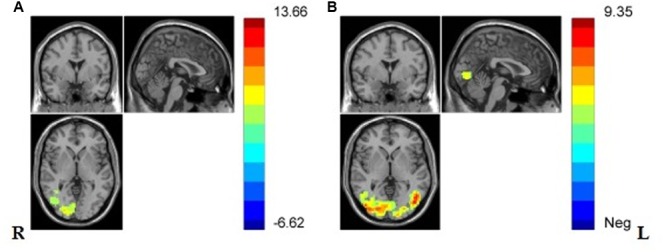
**(A)** Brain regions showing significant activation while viewing painful stimuli compared with non-painful stimuli in NG in lateral middle occipital gyrus, lateral V2, right middle temporal gyrus, lateral fusiform gyrus. (*p* < 0.001, Alphasim corrected; *k* > 1138). **(B)** Brain regions showing significant activation while viewing painful stimuli compared with non-painful stimuli in VG in lateral occipital gyrus, lateral fusiform gyrus, left V2, and right middle temporal gyrus (*p* < 0.001, Alphasim corrected; *k* > 1132).

And one sample *t*-test in VG and NG was separately conducted and the results showed that, in both groups, regions in lateral occipital gyrus, lateral fusiform gyrus, right middle temporal gyrus, and the Secondary visual cortex (V2) were significantly activated (see **Figures 4A,B** and **Table 2**).

**Table 2:** Brain regions showing significant activation in lateral occipital gyrus, lateral fusiform gyrus, left V2, and right middle temporal gyrus in VG, and significant activation in lateral occipital gyrus, lateral fusiform gyrus, right lingual gyrus, left V2, and right middle temporal gyrus while viewing painful stimuli compared with non-painful stimuli (*p* < 0.001, Alphasim corrected; *k* > 1132 in VG and *k* > 1138 in NG).

Conditions	Region of activation	Lat.	MNI			*t-*value	*k*
			*x*	*y*	*z*		
NG	Middle occipital gyrus	R	21	–96	12	13.66	367
	Middle occipital gyrus	L	–42	–87	0	6.32	476
	Secondary visual cortex (V2)	R	36	–87	9	8.26	102
	Secondary visual cortex (V2)	L	–36	–9	0	5.92	116
	Middle temporal gyrus	R	51	–69	12	4.67	99
	Fusiform gyrus	R	36	–48	–15	6.28	249
	Fusiform gyrus	L	–27	–60	–15	7.53	199
VG	Middle occipital gyrus	L	–45	–75	0	7.89	442
	Middle occipital gyrus	R	45	–81	0	7.53	365
	Fusiform gyrus	L	–48	–66	0	8.30	195
	Fusiform gyrus	R	30	–66	–12	7.66	362
	Secondary visual cortex (V2)	L	–12	–78	–9	7.09	268
	Middle temporal gyrus	R	48	–63	–3	6.21	93

Since the focus of our study was to explore how previous exposure to video game violence influenced participants’ empathic responses, we examined the activation of brain regions showing differences between the two groups. There is no significant difference between VG and NG (*p* < 0.001, Alphasim corrected).

## Discussion

The goal of our study was to explore the influence of previous exposure to video game violence on neural empathic responses to the pain of others. fMRI results showed that there is significant difference between viewing painful pictures of others and viewing non-painful pictures, which has also been proved separately in VG and NG. While further examination didn’t show that the empathic neural pattern is different between groups.

Consistent with previous fMRI studies on empathy for pain (e.g., Jackson et al., 2005; Cheng et al., 2008; Nummenmaa et al., 2008; Akitsuki and Decety, 2009; Guo et al., 2012, 2013), the present study found that viewing painful pictures activated many empathy-related regions in the (PVG + PNG) - (NVG + NNG) contrast.

Unlike the linguistic function that has been generally hitherto acknowledged, the supramarginal gyrus is also closely linked to empathy. The supramarginal gyrus is part of the somatosensory association cortex, which is involved in perception of space and limbs location and a part of the mirror neuron system (Carlson, 2012). It has been proved that supramarginal gyrus, especially the right supramarginal gyrus (rSMG) is significantly associated with self-other distinction, the crucial part of the theory of mind (ToM), attributing to the self-other distinction during empathy (Hoffmann et al., 2015). Empathy involves sharing the emotional state of others and being aware of the state both of self and others (Singer and Lamm, 2009). Failure of self-other distinction during empathy results in egocentric emotional responses and deficits in ToM (Hoffmann et al., 2015). Silani et al. (2013) found that overcoming emotional egocentricity bias in empathic judgment is associated with increased activation in the rSMG. What’s more, a research conducted by Lang et al. (2011) found the same rSMG activation to emotional exclamations of others’ pain. This is consistent with what we had expected, as viewing others in pain will activate the regions related to empathy. The occipital gyrus are mainly associated with visual processing (Berlucchi, 2014). It has been proved that the inferior occipital gyrus plays an important part in identifying emotionally important visual clues, and viewing unpleasant pictures can significantly activate the left inferior occipital gyrus compared to the neutral situations (Geday et al., 2003). What’s more, the posterior fusiform and inferior occipital gyrus were assumed as the core regions in identifying emotionally important visual clues (Geday et al., 2003). The present study also found that participants viewing painful pictures had stronger activation in the inferior parietal lobule than those viewing non-painful pictures. The inferior parietal lobule has a critical function in distinguishing between self-produced actions and actions generated by others (Decety and Jackson, 2004; Lamm et al., 2008). A previous fMRI study demonstrated that higher activation of this region reflects less self-other overlap, which leads to greater accuracy during social perception (Lawrence et al., 2006). Another significantly activated region is the lateral fusiform, which is known as a key region related to facial perception. However, it has been proved that fusiform gyrus is associated with the processing of “ToM” and so is empathy (Castelli et al., 2000; Gallagher et al., 2000; Moll et al., 2002). It can be modulated by emotional valence, and it has been proved that right fusiform gyrus was more active than the left during emotional processing (Geday et al., 2003). This is consistent with our findings, which suggest that exposure to painful pictures will induce the emotional response and the unpleasant pictures are more arousing.

It should be noted that there are no significant difference in the full factorial design in [(PVG - NVG) - (PNG - NNG)]. This may suggest that there is no deficit in the neural responses of empathy for pain in individuals with VVG experience, being inconsistent with some extant studies (e.g., Funk et al., 2004; Anderson et al., 2010; Strenziok et al., 2011; Zhen et al., 2011; Montag et al., 2012; Guo et al., 2013), which all suggest that long-term exposure to media violence has a desensitization effect. However, Decety et al. (2009) showed that youth with aggressive conduct disorder do not have a deficit in empathy and may have an atypical pattern of neural response while viewing others in pain. Similarly, a survey conducted by Collins and Freeman (2013) found no difference in empathy between gamers and non-gamers. This can be seen from the painful pictures and non-painful pictures contrast both in VG and NG. The brain activation in both VG and NG showed similar pattern when viewing painful pictures compared to viewing non-painful pictures. The lateral fusiform gyrus was activated in both groups, which is important during empathy.

This may indicate that long-time exposure to VVGs is not strongly associated with desensitization to violence, especially pain empathy to others. This is supported by researches conducted by Szycik et al. (2016, 2017). In their researches, the positive, negative, and neutral pictures were displayed and the fMRI data was collected. Repeated experiments proved that there was no evidence for a neural desensitization in the processing of emotionally salient stimuli, same as our research findings. Taking all the findings together, it is necessary for us to rethink the desensitization hypothesis. The catalyst model proposed by Ferguson et al. (2008) pointed that, just like competition, playing VVGs is the result of attacking intention, not the cause of it. In this case, VVGs are not significantly relevant to aggressive behaviors. At the same time, the catharsis theory of playing contends that playing VVG, especially action game, provide a way to drain the aggressive emotion and energy off, rather than increasing the aggressive belief. After enjoying themselves immersing in the games, the nervous feelings and extra energy were consumed, players are used to feeling entirely free from worry. Compared to researches based on self-report measures, neuropsychological researches are definitely more valid to testify the long-term effect.

This research was based on the comprehensive view of VVG effect without certain bias and based on what was shown in this study, it could be suggested that our research is more objective and convincing. However, our study also has some limitations and there are areas that need further exploration. The present study did not measure sensitivity to pain, so we cannot rule out the possibility that some of our findings were influenced by individual differences among the participants. On the other hand, although there were no gender-related differences on empathy shown in the present study, it still may be caused by the gender distribution. It should be noted that only males were examined in our research and the research is suitable when the participants are confined to males. Further studies should pay attention to gender distribution. Furthermore, unknown variations or inconsistencies in the functions of some brain regions and neural circuits might explain the observed activations in some brain regions. Exploring the influence of VVGs on cognitive empathy and emotional empathy separately may provide the topic with more precise findings.

## Conclusion

The observation that there were no significant differences between VG and NG suggests that individuals with VVG exposure may not have a deficit in their capacity for empathy. The differences in empathy for pain between individuals with VVG experience and non-VVG experience indicated that the desensitization effect of VVGs is not significant.

## Compliance With Ethical Standards

All procedures performed in studies involving human participants were in accordance with the ethical standards of the institutional and/or national research committee and with the 1964 Helsinki declaration and its later amendments or comparable ethical standards. Written informed consent was obtained after detailed explanation of the study protocol, which was approved by the Ethics Committee of Southwest University. The Institutional Review Board at Southwest University (SWU) in Chongqing, China approved this consent procedure. Written informed consent was obtained from all participants. The Institutional Review Board at SWU approved all procedures. Informed consent was obtained from all individual participants included in the study.

## Author Contributions

Conceived and designed the experiments: XG and LW. Performed the experiments: XG, CL, and WP. Analyzed the data: CL, XG, WP, and MY. Wrote the paper: WP, XG, LW, and CL. Editing and Revisions: XG, AC, CL, and WP.

## Conflict of Interest Statement

The authors declare that the research was conducted in the absence of any commercial or financial relationships that could be construed as a potential conflict of interest.
